# Modulating mental state recognition by anodal tDCS over the cerebellum

**DOI:** 10.1038/s41598-022-26914-4

**Published:** 2022-12-30

**Authors:** Silvia Clausi, Michela Lupo, Giulia Funghi, Alessia Mammone, Maria Leggio

**Affiliations:** 1 Ataxia Laboratory, Fondazione Santa Lucia IRCCS, Via Ardeatina, 306 Rome, Italy; 2 Klinikos—Center for Psychodiagnostics and Psychoterapy, Viale delle Milizie, 38, Rome, Italy; 3 Center for Mind/Brain Sciences CIMeC, University of Trento, Corso Bettini, 31, Rovereto, Italy; 4 National Institute for Infectious Diseases Lazzaro Spallanzani - IRCSS, Via Portuense, 292 Rome, Italy; 5 Department of Psychology, Sapienza University of Rome, Via Dei Marsi, 78, Rome, Italy

**Keywords:** Neuroscience, Psychology

## Abstract

Increasing evidence from neuroimaging and clinical studies has demonstrated cerebellar involvement in social cognition components, including the mentalizing process. The aim of this study was to apply transcranial direct current stimulation (tDCS) to modulate cerebellar excitability to investigate the role the cerebellum plays in mental state recognition. Forty-eight healthy subjects were randomly assigned to different groups in which anodal, cathodal, or sham tDCS (2 mA for 20 min) was delivered centering the electrode on the vermis to stimulate the posterior portion of the cerebellum. The ability to attribute mental states to others was tested before and after tDCS using a digital version of the 'Reading the Mind in the Eyes test', which includes visual perceptive and motor stimuli as control conditions. Correct response and reaction times (RTs) were recorded. The results revealed a significant reduction in RTs between the baseline and post-stimulation sessions after cerebellar anodal tDCS only for mental state stimuli (Wilcoxon test *p* = 0.00055), whereas no significant effect was found in the cathodal or sham conditions or for visual perceptive and motor stimuli. Overall, our study suggests that cerebellar anodal tDCS might selectively improve mental state recognition and constitute an effective strategy to positively modulate the mentalizing process.

## Introduction

Over the last decades, the cerebellum has been recognized as a critical structure not only in motor control but also in cognitive and affective functioning^1–6^. More recently, increasing evidence has extended the role of this brain region in the social cognition domain^7–11^. Specifically, several neuroimaging studies have shown cerebellar activation in social cognition tasks^12–15^ and its functional connections with brain regions belonging to the ‘social brain network’ (i.e., the default mode network)^16–21^.

Clinical studies in patients with cerebellar damage demonstrated specific social cognition difficulties, including impairments in basic emotions and mental state recognition^3,11,22,23^, suggesting that alterations in specific cerebellar regions could be responsible for the corresponding neurobiological underpinnings^22^. Very recently, Frosch and colleagues^8^ described the literature regarding cerebellar contributions to social cognition and adaptive prediction in individuals with and without autism.

The emerging idea is that the cerebellum is a good candidate for the predictive function that allows humans to be more adaptive in social interactions^24^ thanks to its neuroanatomical connections with the limbic and associative areas^25–27^.

In this view, the cerebellum might act in the social cognitive domain as a modulator and optimizer of the projection brain areas activity, in a very similar way in which it does in the motor and cognitive domains^4,7,11,17^.

Despite the data reported above, the role played by the cerebellum in social behavior still needs to be better defined. To address this issue, it is crucial to exclude possible confounding effects resulting from to other methodological approaches. For instance, in functional magnetic resonance imaging (fMRI) studies, cerebellar activation during social cognition tasks is often detected together with other brain regions^12,17,18,20^ making it difficult to test its role in specific aspects of information processing. Moreover, cerebellar damage does not often occur in isolation in patient studies^28,29^, making conclusions about direct region-specific effects difficult to draw. Finally, the life changes associated with cerebellar disease onset may themselves produce alterations in social behavior, affecting everyday life as well as compliance with pharmacological and rehabilitative treatments^30^.

In this framework, brain neuromodulation techniques, including transcranial direct current stimulation (tDCS), represent a very advantageous approach to study cerebellar involvement in specific social behavior processes^31^.

Indeed, tDCS is a noninvasive brain stimulation technique used to pass a direct current through the brain of subjects via surface electrodes fixed to the head. It allows in vivo manipulation of neuronal excitability in humans in a polarity-specific manner and induces functional changes in the cerebral and cerebellar cortices^32,33^. This increasingly popular neuroscientific technique offers the opportunity to investigate the behavioral consequences of regional reduction or enhancement in neuronal excitability in healthy individuals^34–38^. Unlike patient studies, the noninvasive stimulation allows us to investigate the role of a specific brain region in behavioral tasks without confounding factors, such as pharmacological treatment, or concomitant damage to other cerebral brain regions^39^**.**

While it is most frequently applied to the cerebral cortex, tDCS was recently used to modulate cerebellar excitability^37,38^. Indeed, in recent years several modeling studies have investigated the distributions, strength and electrode montage-dependent variability in electric fields induced by cerebellar tDCS^40–42^, and freely available pipelines have been presented to optimize the stimulation of cerebellar lobules involved in different cognitive and motor functions^43,44^. These studies demonstrated that the electric field generated during tDCS when applied over the cerebellum effectively reaches its posterior portion with relatively little functional spread to neighboring regions^45^ because of the location of the cerebellum in the posterior cranial fossa.

Currently, this noninvasive brain stimulation technique is providing greater insight into the contribution of the cerebellum in cognitive and social-affective domains^31,46,47^.

Specifically, a recent study explored the effects of cerebellar tDCS on the prediction of social and physical events^48^, showing specific involvement of the cerebellum in forming expectations related to social events, but not to physical events. Moreover, a previous study by Ferrucci and colleagues^49^ showed an effect of both anodal and cathodal cerebellar tDCS on the recognition of basic emotions. However, to the best of our knowledge, no studies have used tDCS applied over the cerebellum to investigate its role in the mentalizing process.

A fundamental aspect of social cognition is the capacity to estimate the mental state of other people in order to understand and predict their behavior. This ability is known as the Theory of Mind (ToM)^50,51^, or mentalizing process, and implies both lower-level processes based on action recognition and “emotional contagion^52^” and a higher-level conceptual capacity to adopt the perspective of others to infer their mental state^53^.

Since in mindreading the first contact between agents is the eyes^54^, a task frequently used in clinical and experimental studies to investigate this ability is the Reading the Mind in the Eyes Test (RMET), which requires subjects to “tune in” to the mental state of the actor’s eye expression at a rapid and automatic level to make the correct choice^55^.

Indeed, although very recent studies hypothesized that this task is likely to assess lower-level processes such as perceptual emotion recognition rather than genuine theory-of-mind abilities (mentalizing criteria)^56,57^, the RMET remains the most used test of theory of mind.

Following the original Baron-Cohen’s description^55^, the RMET involves the first stage of ToM: attribution of a relevant mental state (e.g., compassion), regardless of its content (e.g., compassion for her mother’s loss). This task has been shown to activate a key mentalizing region in the cerebral cortex, the temporoparietal junction^58^. Interestingly, in line with findings by Hoche and colleagues^23^, in recent studies, we found impaired performance in RMET in patients with cerebellar pathology and autism^3,22,59^. A significant correlation has been shown between RMET scores and indices of microstructural alteration in the superior cerebellar peduncles and reduced cerebellar gray matter volumes in patients affecterd by spinocerebellar ataxia type 2^22^.

Thus, we speculated that the structural alterations in posterior portions of the cerebellum (i.e., Crus I/II), which are known to be linked to advanced ToM features^18,60^, may structurally and functionally affect key mentalizing areas in the cerebral cortex, leading to ToM impairments in individuals with cerebellar pathologies or autism.

In the present study, we combined cerebellar tDCS with an ad hoc digital version of the RMET to further understand cerebellar involvement in the first stage of the mentalizing process.

We started from the idea that the cerebellum acts in the domain of mentalization through its interaction with the brain areas involved in such processes^17–20^. Therefore, we used an extracephalic montage in which the active electrode was centered over the cerebellum (median line, 2 cm below the inion) and the reference electrode over the right deltoid muscle to stimulate the posterior cerebellum in line with the modeling study by Parazzini and colleagues^45^.

This montage avoids confounding effects because an electrode with opposite polarity placed over the scalp could interfere with the activity of brain areas belonging to such networks, not allowing us to isolate the effect of cerebellar stimulation on task performance^40,49,61^.

Based on the possible excitatory and inhibitory influences of anodal and cathodal tDCS on cerebellar cortical excitability we also hypothesized that anodal and cathodal polarity increases and reduces, respectively, optimization of the mentalizing process. We tested this hypothesis in 48 healthy individuals using a randomized, sham-controlled, double-blind, between-subjects design.

## Results

None of the participants reported adverse effects of tDCS as evidenced by an ad hoc questionnaire administered at the end of every experimental session.

No differences between groups were found with respect to age, education, or the Edinburgh Handedness Inventory (EHI)^62^ as assessed by the Kruskal–Wallis test. A lower proportion of females was assigned to the anodal group [5/16 (36%)] compared to the cathodal group [9/16 (56%)] and to the sham group [12/16 (75%)], as tested by the chi-square test (see Table 1). For each group, no differences between baseline and post-stimulation sessions were detected in any of the VAS scores^63^ (anxiety, mood and fatigue) (Table 2).

**Table 1:** Demographic characteristics of the three groups.

Group	N	Gender (F/M)	Age mean (SD)	EHI mean (SD)	Education mean (SD)	Raven’47 mean (SD)
Anodal	16	5/11	25.68 (6.78)	16 (2)	85.56 (11.47)	29.71 (1.88)
Cathodal	16	9/7	30.75 (11.49)	14.87 (2.06)	71.68 (49.31)	29.79 (1.06)
Sham	16	12/4	23.31 (2.86)	15.43 (1.86)	73.43 (49.14)	29.44 (1.57)
Total	48	26/22	25.71 (6.32)	15.45 (1.96)	70.43 (49.43)	29.66 (1.5)
*p* value		**0.044**†	0.18*	0.38*	0.70*	0.89*

*F*: Female, *M*: Male, *SD*: Standard deviation, *EHI*: Edinburgh Handedness Inventory.

^†^*p* value refers to Chi-Square Test; *p value refers to Kruskall–Wallis Test.

Significant values are in bold.

**Table 2:** Results obtained by each group at the baseline and after the tDCS session. The data are presented as means and standard deviation (SD). VAS scores for anxiety, mood and fatigue are reported in centimetres. For the RMET the accuracy is reported as number of correct response and the reaction times in milliseconds. Wilcoxon test *p *values ≤ 0.005 are reported in bold. A significant decrease of the RTs between the baseline and post-stimulation sessions is evident only for the mental state stimuli after the anodal cerebellar tDCS.

		Stimulation	Pre	Post	Z	*p* value
VAS scores	Anxiety	Anodal	3.1 (0.6)	2.8 (0.7)	0.396	0.692
		Cathodal	2.4 (0.45)	1.7 (0.35)	1.127	0.260
		Sham	3.8 (0.6)	2.6 (0.5)	1.490	0.136
	Mood	Anodal	7.6 (0.4)	6.3 (0.5)	0.113	0.910
		Cathodal	6.3 (0.6)	6.8 (0.6)	− 0.717	0.474
		Sham	6.2 (0.7)	6.5 (0.6)	− 0.282	0.777
	Fatigue	Anodal	4.2 (0.5)	3.3 (0.5)	1.150	0.250
		Cathodal	3.7 (0.9)	3.9 (0.7)	− 0.245	0.806
		Sham	3.5 (0.7)	3.8 (0.6)	− 0.603	0.546
RMET—Accuracy	Mental state	Anodal	25.5 (2.85)	26.7 (2.26)	− 1.008	0.313
		Cathodal	25.9 (2.35)	25.7 (2.6)	0.477	0.633
		Sham	26.9 (2.58)	27.4 (2.91)	− 0.570	0.568
	Visual Perception	Anodal	35 (0.82)	35.2 (0.93)	− 1.035	0.300
		Cathodal	35.2 (0.6)	35.4 (1.09)	− 1.403	0.160
		Sham	35 (0.89)	35 (0.89)	0	1.000
	Visuomotor	Anodal	35.5 (0.73)	35.2 (0.58)	1.31	0.188
		Cathodal	35.9 (0.34)	35.8 (0.4)	0.449	0.653
		Sham	35.8 (0.4)	35.3 (0.87)	1.92	0.054
RMET—RTs (ms)	Mental state	Anodal	3446 (697)	3007 (629)	2.546	**0.00055**
		Cathodal	3805 (649)	3460 (631)	1.470	0.0141
		Sham	3638 (532)	3414 (626)	1.149	0.25
	Visual Perception	Anodal	1009 (207)	924 (182)	1.131	0.25
		Cathodal	1178 (247)	1073 (198)	1.074	0.28
		Sham	1156 (238)	1054 (143)	1.413	0.16
	Visuomotor	Anodal	689 (93)	641 (61)	1.622	0.105
		Cathodal	768 (173)	728 (137)	0.603	0.546
		Sham	750 (99)	703 (87)	1.320	0.187

*VAS*: Visual analogue scale, *RMET*: Reading the mind in the eyes test, Accuracy = number of correct responses.

At baseline, the Kruskal–Wallis one-way analysis of variance showed no differences among the three groups for the correct response (MS stimuli: H = 1.39; *p* = 0.49; V-P stimuli: H = 0.82; *p* = 0.66; V-M stimuli: H = 3.48; *p* = 0.17) or RTs in each stimulus type (MS stimuli: H = 2.99; *p* = 0.22; V-P stimuli: H = 5.84; *p* = 0.05; V-M stimuli: H = 2.58; *p* = 0.27).

Moreover, no differences in the number of correct responses were found between baseline and post-stimulation sessions in any group or for any stimuli, as assessed by the Wilcoxon test (see Table 2 for statistical details).

Notably, an improvement in the processing of mental state stimuli was evident after anodal stimulation, as shown by the significant decrease in RTs between the baseline (mean ± standard deviation: 3446 ± 697) and post-stimulation (mean ± standard deviation: 3007 ± 629) sessions for MS stimuli (Wilcoxon test, *p* = 0.00055).

**Figure 1:**
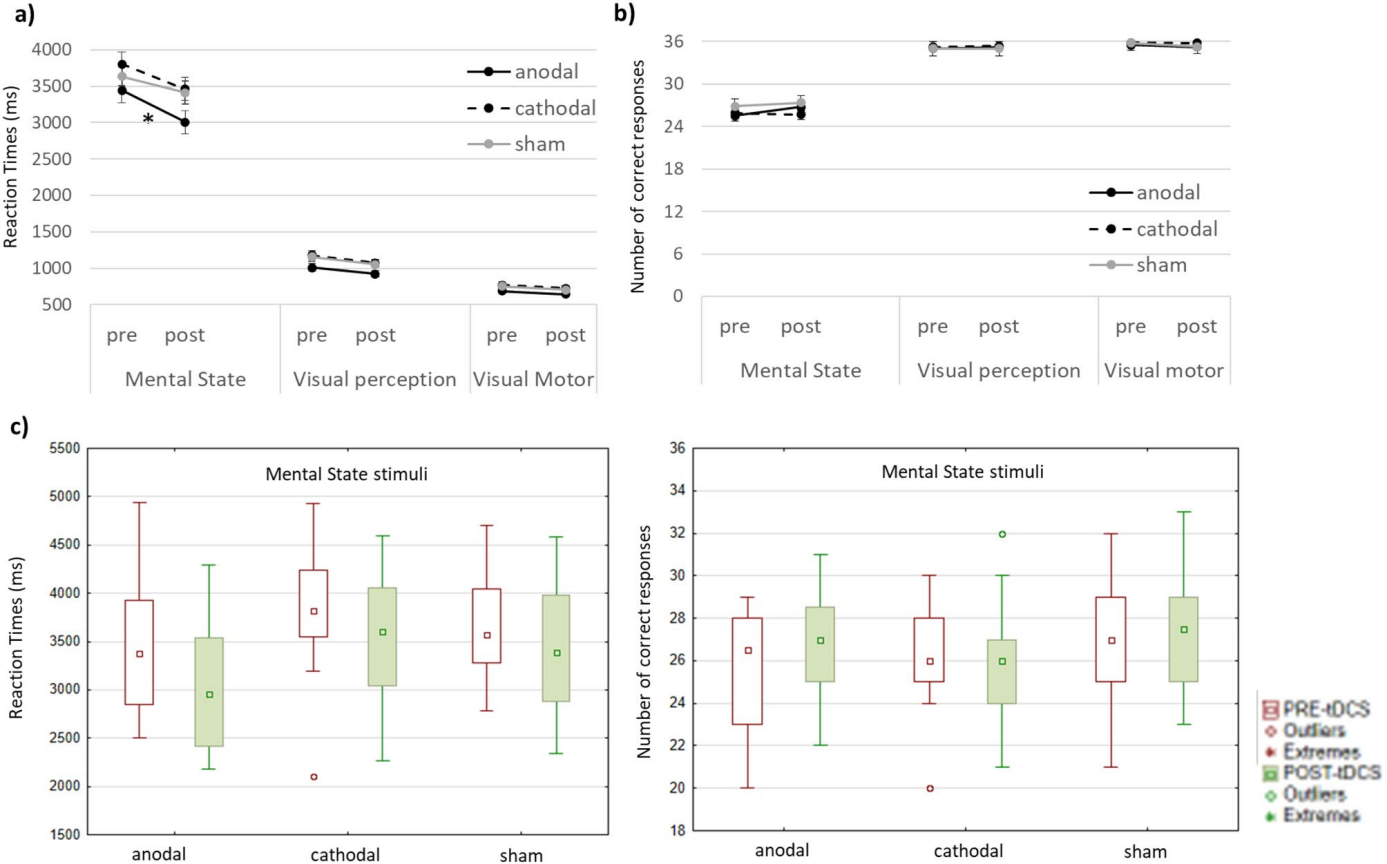
Reading the Mind in the Eyes Test results. Graphs show the mean reaction times (in milliseconds) (**a**) and the number of correct responses (**b**) for each group and stimuli type before and after tDCS stimulation. Standard Error is reported (**a, b**). *p value < 0.005. Regarding the mental state stimuli, the results for each group are plotted as median before (in red) and after stimulation (in green) (**c**). Box: 25–75%; Whisker: non-outlier range.

A reduction in RTs between the baseline (mean ± standard deviation: 3805 ± 649) and post-stimulation (mean ± standard deviation: 3460 ± 631) phases was also evident in the cathodal group but was not significant after Bonferroni correction (Wilcoxon test, *p* = 0.01). No significant differences in RTs were observed between baseline and post-stimulation sessions for V-P or V-M stimuli in either the anodal or cathodal group. Again, no significant differences were found in RTs of the sham group for any stimulus type. The RMET results are detailed in Table 2 and illustrated in Fig. 1.

## Discussion

In recent years, noninvasive brain stimulation has been increasingly used to study cerebellar involvement in motor control and cognitive functions^37^. However, only a few studies have used this approach to investigate the cerebellar role in social behavior^31^ focusing on basic emotion recognition by the processing of facial expressions^49,64^ and on the prediction of social events^48^. In particular, Ferrucci and colleagues^40^ used a standard facial emotion recognition task, focusing on basic emotions such as anger, happiness, and sadness, while Oldrati and colleagues^48^ investigated cerebellar involvement in forming expectations related to the social events.

Unlike the abovementioned studies, we used the RMET as a measure of adult ‘mentalizing’. As described by Baron-Cohen et al.^55^, this advanced theory of mind test involves matching terms describing complex mental states to pictures showing only the eye region. In particular, to perform the task correctly subjects have to “match the eyes in each picture to examples of eye-region expressions stored in memory and seen in the context of particular mental states to arrive at a judgment of which word the eyes most closely match^55^”. This task involves only the first stage of attribution of the theory of mind, namely attribution of the relevant mental state, but not inferring the content of that mental state which represents the second level.

The present study extends the current knowledge in this field, since it demonstrates for the first time the causal effect of cerebellar neuromodulation on the first stage of the mentalizing process.

Specifically, we found that the anodal tDCS delivered over the posterior cerebellum enhanced the processes subtending the ability to recognize the mental state of another person as measured by RMET. We observed the same tendency when cathodal polarity was applied, even though it did not reach statistical significance after Bonferroni correction for multiple comparisons.

Interestingly, the effect on mental state processing was not correlated with a general speeding up, given that cerebellar stimulation did not modify the elaboration speed in terms of RTs when the participants processed perceptual or visuo-motor stimuli.

In our task, the subjects had to read and correctly comprehend the single adjectives, abilities in which a role for the cerebellum has been described^65^. However, previous studies have shown that anodal cerebellar tDCS does not modulate reading ability and language processing performance in healthy individuals^66,67^.

Moreover, in agreement with previous cerebellar neuromodulation studies^49,64^, we did not observe any changes in anxiety, mood or fatigue after cerebellar stimulation.

Although we started from the assumption that increased and reduced cerebellar excitability (due to anodal and cathodal stimulation) could increase and reduce, respectively, optimization of the mentalizing process, we found a similar effect for the two polarity types in the present study. This observation agrees with the lack of polarity-specific tDCS-induced changes observed in cognitive/affective tasks^47,49^.

One possible explanation for the lack of polarity specificity of cerebellar tDCS comes from general physiological mechanisms that have been known for years, by which a functional inhibition/disruption in any excitable tissue can be obtained with both depolarization and hyperpolarization^68^. For instance, classic neurophysiological experiments demonstrated that axonal conduction can be blocked, even for several hours, by depolarization ("depolarizing" block) and by hyperpolarization ("hyperpolarizing" or "anodal" block), leading to the same decreased excitability of the stimulated tissue^68^.

This lack of polarity specificity could also be applied to the cerebellum, since both hyperpolarization and depolarization of the Purkinje cells can lead to a block of cerebellar cortex excitability^45,69^. Furthermore, the effect on cerebellar excitability modulation could be even more complex, considering the cerebellar cortico-nuclear interactions. Specifically, Purkinje cells are well known to inhibit the deep cerebellar nuclei, which in turn have an excitatory action on the cerebral cortex through the cerebello-thalamo-cortical pathway^25,70^. Therefore, at baseline, the Purkinje cells exert an inhibitory tone on the projection brain areas, namely cerebellar brain inhibition^71,72^^.^ Accordingly, any changes in Purkinje cell excitability, either positive or negative, might significantly influence the efficiency of information transmission to the projection brain areas^73^, through the action exerted on the deep cerebellar nuclei. Therefore, in our experiment, blockade of cerebellar cortex excitability might lead to disinhibition of the deep cerebellar nuclei, facilitating the activity of cerebral areas involved in the mentalizing process with which the cerebellum is connected^25,27,70^. In this way, cerebellar tDCS improves the response efficiency to mental state stimuli.

### The cerebellar role in the mentalizing process

Our study should be seen in the context of the increasing interest of the scientific community in the key role of the cerebellum in social behavior^7^.

A main component of an adaptive social behavior is the ability to infer others’ mental states, in order to anticipate future interactions^74^. For a long time, the primary neurobiological underpinning of this dynamic ability has been localized in the cerebral cortex, including the brain areas known as the mentalizing network^75,76^. More recently, the focus has been extended to the cerebellum^7^ and fMRI studies have shown that specific cerebellar areas are activated during mirroring and mentalizing tasks^12,76^, leading to a description of new specific cerebro-cerebellar pathways.

More specifically, studies on healthy individuals and clinical populations have identified the posterior cerebellar region (i.e., Crus II) as a key area within the mentalizing network^7,18^. This cerebellar area was also identified as functionally specialized in social mentalizing and emotional self-experiences, showing a distinct functional connectivity with cortical mentalizing areas during mentalizing tasks involving others’ beliefs and traits^7^.

Consistent with these findings, in the present study we used an extracephalic montage to target the posterior cerebellum as described in the modeling study by Parazzini and colleagues^45^, avoiding direct interference with the activity of other brain areas belonging to the mentalizing network and allowing us to isolate the effect of cerebellar stimulation on task performance^32,33,40,49,61^.

What we observed was that tDCS over the posterior cerebellum induced changes in the first stage of the mental state recognition process as measured by the RMET.

Interestingly, our results not only provide new evidence on the direct link between cerebellar functioning and the mentalizing process but also add new insights into the possible mechanisms that are implemented by the cerebellum to modulate the mentalizing process.

As reported in the introduction, we started with the idea that the cerebellum acts in the social domain in the same way as it does in the sensorimotor domain. Indeed this brain structure performs a predictive action based on forward internal models, signalizing deviations from the attended outcomes to the projection cerebral areas^4^. This operational mode is implemented by the constant modulation that the cerebellum exerts on brain regions involved in the processing of information related to specific functional domains^25^.

Accordingly, in the case of the RMET used in the present study, the cerebellum acts to implicitly match the external information, such as the expression of the eyes, with the internal model of eye region expression linked to previous emotional experiences to guarantee an immediate judgment about the mental state of others.

When cerebellar excitability is modulated by tDCS, the required fast and continuous exchange of information between the external stimuli and the internal model is facilitated, thus speeding up the mindreading processes. Our results are also in line with recent studies in patients with cerebellar pathology, in which impairment in mental state recognition measured by the RMET was reported^3,23^. This impairment has been linked to the absence of cerebellar modulation action on the cerebral cortex projection areas involved in mentalizing. In fact, when cerebellar damage is present, the required fast and continuous exchange of information between the external stimuli and the internal model might be affected, thus interfering with the process speed^3,22^.

### Clinical implications and future directions

The present study describes new possibilities for the application of cerebellar tDCS, not only in patients with cerebellar damage but also in those clinical conditions in which this brain structure is implicated. Indeed, as extensively reported in the literature, the cerebellum has been described as involved in the pathogenesis of psychiatric disorders (i.e., schizophrenia) and neurodevelopmental conditions (i.e., autism spectrum disorders) characterized by social behavior difficulties^77,78^. Structural and functional cerebellar alterations have been reported in these conditions^77,79–81^. In line with this, knowing more about the cerebellar tDCS-induced changes in mental state recognition ability would aid in developing new therapeutic protocols in these patient populations^82^.

The primary strength of this approach is that it does not generate discomfort and can be easily combined with cognitive-behavioral therapy in those pathological conditions that present with pharmacological treatment resistance^83^. All in all, noninvasive cerebellar stimulation may represent a promising strategy for improving residual cerebellar circuit functioning and as a complementary tool for rehabilitation protocols in patients with cerebellar dysfunction^84^. However, we have to consider that the after-effects of tDCS over the cerebellum are highly variable among individuals. Additionally, the stimulation effects could be different depending on whether a behavior is tested during (on-line effects) or after (off-line effects) the stimulation session.

These aspects highlight the need to better understand the individual factors that determine the efficacy of this technique (e.g., the baseline neural excitability, cognitive capacity, or personality traits) and to test this cerebellar tDCS protocol in a larger and more homogeneous sample.

Before drawing conclusions, we must consider some limitations.

First, we are aware that the small sample size for each group (n = 16) might affect the statistical power of the analyses. Moreover, the nonnormal distribution of our sample and the tDCS effect variability due to the difference in skin impedance among the participants might limit the power of the present conclusion due to the use of nonparametric statistics. Indeed, it is well known that the efficacy of neuromodulation depends not only on the positioning/design of the electrode montage but also on interindividual variability owing to anatomical differences^41^, as well as neurophysiological ones based on cytoarchitecture^85^.

To obviate this limitation, we applied the Bonferroni correction for multiple comparisons, after which only the significant effect in the anodal condition survived.

Second, the lack of computational modeling does not allow us to predict the electric field induced in our targeted cerebellar areas by the selected tDCS montage. Therefore, future studies should include a specific computational approach to guarantee more accurate targeting of the region of interest.

Finally, the lack of functional neuroimaging administration does not allow validation of the results of the behavioral task to demonstrate the possible cerebellar tDCS effect on cerebello-cortical mentalizing networks. Therefore, studies employing noninvasive brain stimulation with functional imaging^86^ data would improve our understanding of the effect of cerebellar stimulation on participating brain networks.

## Conclusions

The present study demonstrates that anodal cerebellar tDCS enhances the processes underlying the ability to recognize the mental state of another person and that the cerebellum directly contributes to the first stage of mental state recognition. Our findings reinforce and extend current knowledge on the role of the cerebellum in the mentalizing process, and add new insights into the possible application of cerebellar tDCS in the social cognition domain. In this view, the cerebellum could be considered a promising target of noninvasive neurostimulation in the treatment of various impairments of social cognition reported in both neurological and psychiatric disorders associated with cerebellar dysfunction. We believe that these aspects are crucial in clinical practice, and we are confident that our results have clinical and translational potential in terms of treatment implementation.

## Methods

### Participants

Forty-eight tDCS-naïve healthy right-handed volunteers with no history of neurological or psychiatric conditions participated in the study (demographic characteristics are reported in Table 1). The sample size for our between-subjects experiment was determined a-priori considering the mean sample size of previous studies reported in a recent meta-analysis on the tDCS effects on non-motor functions^47^.

Handedness was confirmed using the Edinburgh Handedness Inventory (EHI)^62^. Before recruitment, a neurological objective examination was performed including a questionnaire to verify the suitability for tDCS administration.

None of the participants reported treatments for neuropsychiatric and brain-related disorders or took medications or illicit drugs that could affect the central nervous system. Their intellectual level as assessed by Raven’s 47 progressive matrices was in the normal range (score below the cut-off value of 18.96)^87^.

A double-blinded, randomized, placebo-controlled study was conducted, and each participant was randomly assigned to different groups in which anodal, cathodal, or sham tDCS was delivered over the cerebellum. All groups were well-matched with respect to age, education and intellectual level as reported in Table 1.

A digital version of the RMET^55^ and three self-evaluation ‘Visual Analogue Scale’ (VAS)^63^ assessments for mood, anxiety and fatigue were administered before and 35 min after the end of the tDCS stimulation over the cerebellum, since previous studies showed that the major effect appeared after this time-delay^49,61^^.^ The stimulation type was not known to the experimenter responsible for the testing procedures.

All participants signed an informed consent form in accordance with the Declaration of Helsinki, and the experimental procedures were approved by the Ethical Committee of the IRCCS Santa Lucia Foundation of Rome (Prot. CE/PROG.570).

### Cerebellar tDCS protocol

The real stimulation was delivered by a DC stimulator (BrainSTIM, EMS s.r.l, Bologna) connected to a pair of saline-soaked sponge electrodes (6 × 7) at 2 mA intensity (current density = 0.06 mA/cm2) for 20 min^49^. The active electrode was centered over the cerebellum (median line, 2 cm below the inion) and the reference electrode placed over the right deltoid muscle to stimulate the posterior cerebellum as reported in the modeling study by Parazzini and colleagues^45^.

This extracephalic montage guarantees the highest electric field and current density below the stimulating electrode with only slight spread to other structures (e.g., occipital cortex and brainstem)^45^. Moreover, it has already been used in previous cerebellar tDCS studies to avoid any confounding effects due to the action of the opposite polarity electrode on other brain areas and was found to be effective in modulating facial emotion recognition processing and other cognitive tasks in a sample of healthy adults^40,49,61^.

Stimulation was applied with anodal or cathodal polarity, referring to the electrode placed on the cerebellum or in a placebo mode (sham).

**Figure 2:**
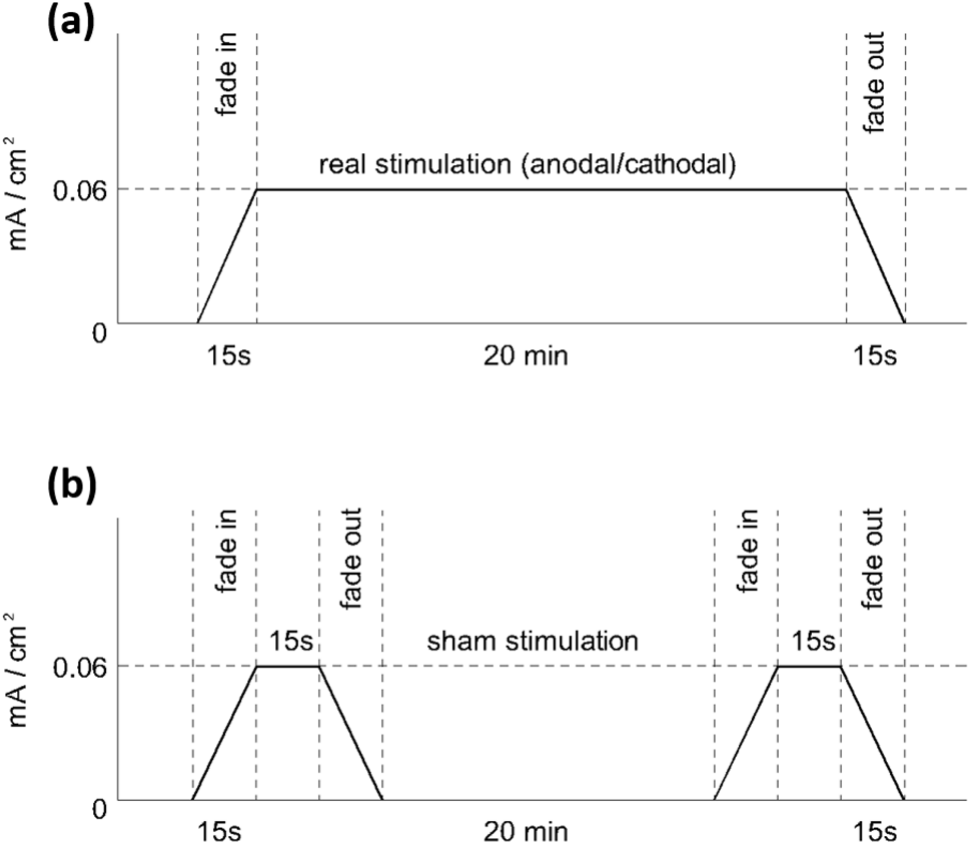
Time course of the tDCS real vs sham stimulation. Schematic depiction of the time course of the real cerebellar tDCS conditions (**a**) and time course of the sham tDCS condition (**b**).

In the real condition, the stimulation was ramped down (15 s) after 20 min of 2 mA tDCS. In the sham condition, the ramp up period was followed by 15 s of real stimulation after which the intensity was ramped down to 0 mA (Fig. 2). This procedure allows the participants to feel the characteristic tingling sensations in the vicinity of the electrodes for a brief period of time, thus enhancing the plausibility of the control condition. In the sham group, half of the participants underwent stimulation with the anodic electrode over the cerebellum, and the other half underwent stimulation with the anodic electrode over the right deltoid.

**Figure 3:**
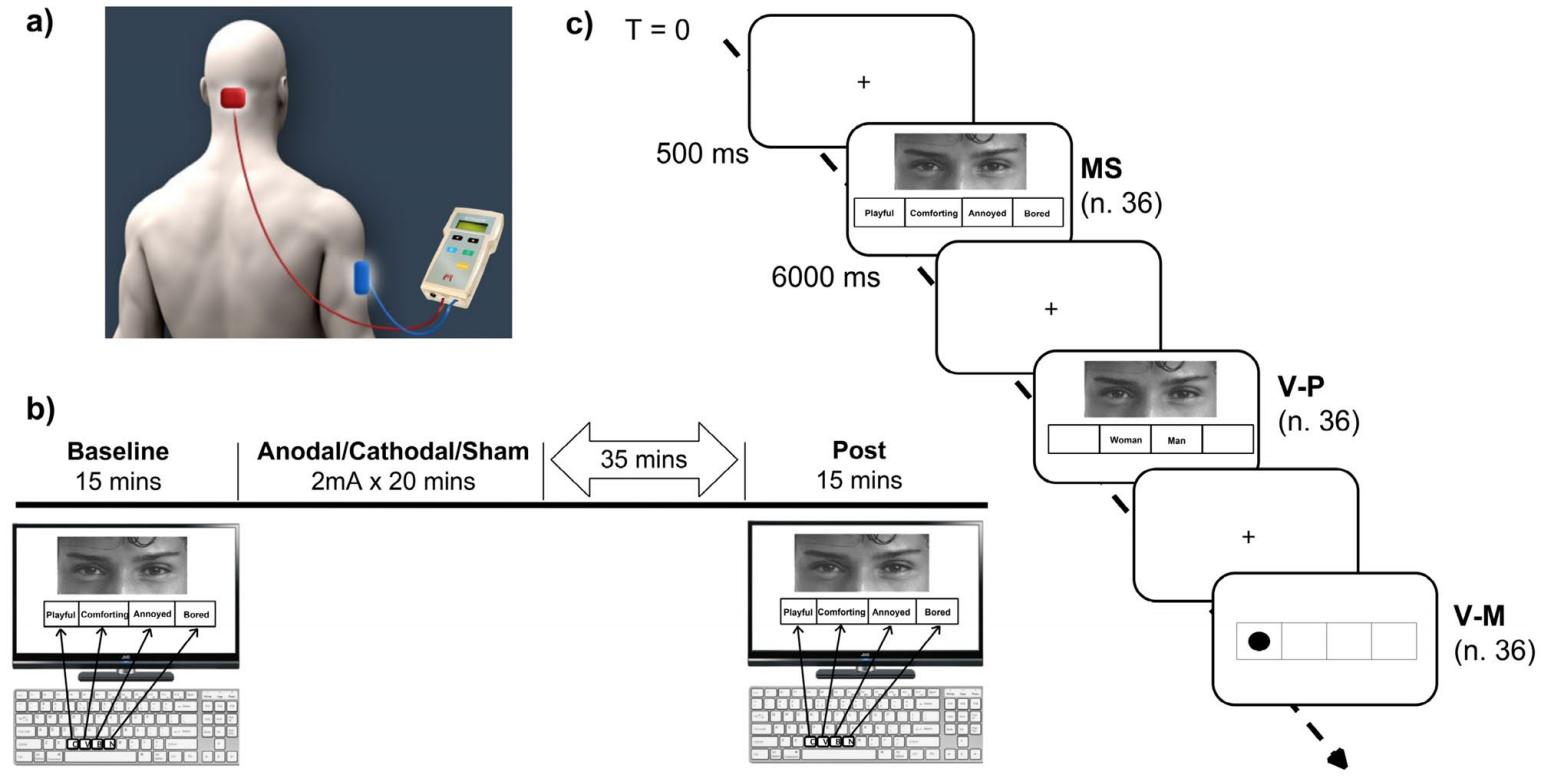
Schematic depiction of the experimental procedure. (**a**) Example of tDCS extracephalic montage in which the anode (red) is centered 2 cm below the inion and the cathode (blue) is placed over the right deltoid. (**b**) Time course of the experimental phases: the behavioral task was administered, before and 35 min after the end of the stimulation session, using a PC with Presentation software, and the correct response and reaction time were recorded; the tDCS session lasted 20 min. (**c**) Stimuli examples of the digital RMET: in the computerized version of the RMET the original photos (MS stimuli) were randomly administered together with two kinds of control stimuli to evaluate visual perception (V-P) (trials = 36) and visual motor (V-M) (trials = 36) factors. The stimuli were administered with an intertrial interval of 6 s and were preannounced by a cross appearing in the middle of the screen for 500 ms. MS = Mental State stimuli; V-P = visual perception; V-M = visual motor. Affinity Photo 1.10 was used to optimize images (https://affinity.serif.com), and Microsoft Office 365 PowerPoint was used to layout the figure.

During the stimulation session, the participants were not involved in specific cognitively demanding activities and the investigator was present throughout the experimental session to check for adverse effects or any technical issues. At the end of every tDCS session, participants completed an ad hoc questionnaire to test for possible adverse effects, including headache, nausea, and impaired balance. Details about the tDCS protocol and montage are also reported in Fig. 3.

### Behavioral tasks

#### Reading the Mind in the Eyes Test (RMET)–digital version

The RMET consists of 36 photographs of the eyes of different actors (19 men and 17 women) illustrating an emotionally charged or neutral mental state (MS–stimuli). As in the original version by Baron-Cohen and colleagues^55^, the subject is required to choose from 4 words (displayed below the photograph) the one that best describes what the person in the photos is thinking or feeling.

In the present study, we used a computerized version of the RMET in which the original photos (MS stimuli) were randomly administered together with two kinds of control stimuli to evaluate visual perception (V-P) (trials = 36) and visual motor (V-M) (trials = 36) factors. All the stimuli were presented on a PC screen (size: 38 cm × 21.6 cm; 17 inches) in Italian ^85^.

In our experiment, subjects sat comfortably approximately 60 cm in front of the PC monitor and used the C, V, B, and N keys of the keyboard to respond. The left-hand middle and index fingers were positioned on the C and V keys, respectively, and the right-hand index and middle fingers on the B and N keys, respectively.

In the MS trials, 4 adjectives appeared in a four-cell grid placed under each photo, and the subject was required to press the key corresponding to the position in which the chosen adjective appeared. In the V-P trials, the words ‘man’ and ‘woman’ appear in two of the four grid cells placed under each photo, and the subject was required to judge the actor’s gender from the eyes, pressing the key corresponding to the position in which the chosen adjective appeared in the grid. For the V-M trials, a black dot appeared in one of the four grid cells, and the subject was required to press the key corresponding to the position in which the dot appeared.

The three types of stimuli were randomly administered with an intertrial interval of 6 s and were preannounced by a cross appearing in the middle of the screen for 500 ms. Overall, the administration of the digital RMET test takes approximately 10 min.

The subject had to respond as quickly and accurately as possible and did not receive error feedback.

The task was administered before and 35 min after the end of the stimulation session using a PC with Presentation software, and the correct response (accuracy) and reaction time (RT) in milliseconds were recorded. The trial structure and examples of stimuli are reported in Fig. 3.

#### Visual analogue scale (VAS)

The VAS^63^ consists of a horizontal line, 100 mm in length, anchored at each end by a word descriptor, and the subject is required to mark the line at the point they felt best represented how they perceived their current state. The VAS score was calculated by measuring the distance from the left-hand end of the line to the point that the subject marked in millimeters.

We used VAS for anxiety (0 mm, no anxiety and 100 mm, the worst anxiety), mood (0 mm, the worst mood and 100 mm, the best mood) and fatigue (0 mm, no fatigue and 100 mm, the highest level of fatigue).

### Statistical analysis

The Shapiro–Wilk test was used to assess our sample distribution, which was not normally distributed (see Table 3 for details). Therefore, nonparametric analysis was performed.

**Table 3:** Results of the Shapiro–Wilk test for the sample distribution.

	Group	Age	Education	EHI	Raven’47
Shapiro–Wilk W	Anodal	0.639	0.792	0.928	0.876
	Cathodal	0.841	0.788	0.645	0.929
	Sham	0.955	0.801	0.515	0.877
	Total	0.758	0.795	0.531	0.892
Shapiro–Wilk p	Anodal	< .001	0.002	0.226	0.034
	Cathodal	0.010	0.002	< 0 .001	0.239
	Sham	0.579	0.003	< 0.001	0.035
	Total	< 0 .001	< 0 .001	< 0.001	< 0.001

*EHI*: Edinburgh Handedness Inventory.

The Kruskal–Wallis one-way analysis of variance with group (anodal N = 16, cathodal N = 16, sham N = 16) as the independent variable was used to test differences in age, education, Raven^87^ and EHI^62^ scores. The chi-square test (N F/M = 26/22) was used to evaluate the association between group and gender.

To exclude significant differences in the performance among the three groups at baseline, Kruskal–Wallis one-way analysis of variance with group as an independent variable was used to compare the accuracy and RT for each stimulus type (MS, V-P, V-M). Statistical significance was considered at *p* < 0.05, and direct comparisons between groups were performed by applying the Bonferroni post hoc correction if necessary.

The Wilcoxon test for matched pairs (dependent samples) was used to assess differences in VAS^63^ scores (anxiety, mood and fatigue) and in accuracy and RT for the different stimuli of the RMET^55^ before and after tDCS, separately for each group. A *p* value ≤ 0.005, as corrected for 9 multiple comparisons by the Bonferroni test, was considered significant. Statistical analyses were performed using SPSS for Windows (version 21.0, Armonk, NY: IBM Corp. Released 2012).

### Ethics approval

Approval of the experimental protocols was obtained from the local ethical committee of the IRCCS Santa Lucia Foundation of Rome (Prot. CE/PROG.570).

### Consent to participate

Written informed consent was obtained from all participants before starting the study.

## Data Availability

The datasets used and analyzed during the current study available from the corresponding author (Dr. Silvia Clausi) on reasonable request.
